# The Interaction between Mushroom Polysaccharides and Gut Microbiota and Their Effect on Human Health: A Review

**DOI:** 10.3390/biology12010122

**Published:** 2023-01-12

**Authors:** Jiahui Zhao, Yixin Hu, Chao Qian, Muhammad Hussain, Shizhu Liu, Anqiang Zhang, Rongjun He, Peilong Sun

**Affiliations:** 1 College of Food Science and Technology, Zhejiang University of Technology, Hangzhou 310014, China; 2 Zhejiang Fangge Pharmaceutical Co., Ltd., Qingyuan 323800, China; 3 Bioactives and Functional Foods Research Center, China National Light Industry, Hangzhou 310014, China; 4 Key Laboratory of Food Macromolecular Resources Processing Technology Research, China National Light Industry, Hangzhou 310014, China

**Keywords:** gut microbiota, mushroom polysaccharides, diabetes mellitus, obesity, non-alcoholic fatty liver disease, inflammatory bowel disease, cancers

## Abstract

**Simple Summary:**

A growing number of studies have shown that mushroom polysaccharides could exert anti-diabetes, anti-intestinal inflammation and antitumor effects by regulating gut microbiota. Thus, the relationship between mushroom polysaccharides and gut microbiota was comprehensively summarized in this review. The vital role of gut microbiota in disease was also emphasized.

**Abstract:**

Mushroom polysaccharides are a kind of biological macromolecule extracted from the fruiting body, mycelium or fermentation liquid of edible fungi. In recent years, the research on mushroom polysaccharides for alleviating metabolic diseases, inflammatory bowel diseases, cancers and other symptoms by changing the intestinal microenvironment has been increasing. Mushroom polysaccharides could promote human health by regulating gut microbiota, increasing the production of short-chain fatty acids, improving intestinal mucosal barrier, regulating lipid metabolism and activating specific signaling pathways. Notably, these biological activities are closely related to the molecular weight, monosaccharide composition and type of the glycosidic bond of mushroom polysaccharide. This review aims to summarize the latest studies: (1) Regulatory effects of mushroom polysaccharides on gut microbiota; (2) The effect of mushroom polysaccharide structure on gut microbiota; (3) Metabolism of mushroom polysaccharides by gut microbiota; and (4) Effects of mushroom polysaccharides on gut microbe-mediated diseases. It provides a theoretical basis for further exploring the mechanism of mushroom polysaccharides for regulating gut microbiota and gives a reference for developing and utilizing mushroom polysaccharides as promising prebiotics in the future.

## 1. Introduction

The gut microbiota is a complex ecosystem, with an estimated 100 trillion gut microbes in adults [1]. Gut microbiota can be divided into six categories: *Bacteroidetes*, *Firmicutes*, *Proteobacteria*, *Actinobacteria*, *Fusobacteria* and *Verrucomicrobia*. Among them, the dominant phyla *Bacteroidetes* and *Firmicutes* account for more than 90% of the gut microbiota [2,3]. Gut microbiota is an important bridge between diet and host health, and plays a crucial role in maintaining homeostasis [4,5]. Meanwhile, gut microbiota is closely related to host metabolism, immune regulation, energy consumption and other physiological processes [6]. A healthy gut microbiota shows diverse species, stable microbiota structure and balanced microecology. Studies have shown that dyshomeostasis of gut microbiota is associated with diseases such as inflammatory bowel disease (IBD), obesity, diabetes mellitus (DM), non-alcoholic fatty liver disease (NAFLD) and carcinomas [7,8,9,10].

Mushrooms are a kind of large fungi, which mainly grow in tropical and humid environments, and have been observed in Asian countries for more than two thousand years. Mushrooms are an important source of natural bioactive ingredients that mainly contain polysaccharides, proteins, vitamins, minerals and dietary fibers [11]. Currently, commonly reported mushrooms mainly include *Ganoderma lucidum* (*G. lucidum*), *Lentinula edodes* (*L. edodes*), *Hericium erinaceus* (*H. erinaceus*), *Auricularia auricular*, *Grifola frondosa* (*G. frondosa*) and *Pleurotus eryngii* (*P. eryngii*). Polysaccharides are the main active ingredients in mushrooms. In recent decades, many studies have shown that mushroom polysaccharides have anti-tumor, anti-inflammatory, antioxidant, anti-diabetes, anti-obesity and other biological activities [12,13,14]. In addition to the above-mentioned biological activities that have been widely reported, the regulation of gut microbiota by mushroom polysaccharides by stimulating the growth of beneficial bacteria has also received extensive attention [15,16,17]. This paper reviews recent developments regarding the regulatory effects of various mushroom polysaccharides on gut microbiota and summarizes the potential mechanism of mushroom polysaccharides to prevent and control diseases through gut microbiota. Additionally, as the biological activity of mushroom polysaccharides is largely affected by their complex structure, it is of great significance to explore the relationship between the structure of mushroom polysaccharides and the regulatory activity of gut microbiota. Consequently, the potential relationship between the structure of mushroom polysaccharides and intestinal flora has been summarized in this paper, which aimed to provide a reference for the development of mushroom polysaccharides in intestinal flora-related diseases.

## 2. Effects of the Structural Characteristics of Mushroom Polysaccharides on Gut Microbiota Diversity

Mushrooms polysaccharides are a natural polymer connected by polyhydroxyaldehyde or polyhydroxyketone through the glycosidic bond; they exert notable bioactivities such as anti-tumor, antioxidant, anti-diabetes, immunomodulation and anti-inflammatory effects [18,19,20,21,22]. Besides, mushroom polysaccharides exert an essential role in regulating the abundance and proportion of gut microbiota closely related to various diseases [23]. Many studies indicated that mushroom polysaccharides such as Wild *morchella* polysaccharides, *Inonotus obliquus* polysaccharides, *Flammulina velutipes* (*F. velutipes*) polysaccharides and *Lentinan* could elevate the relative abundance of *Bacteroidetes* and reduce the relative abundance of *Firmicutes* in the intestinal tract [24,25,26,27]. Supplementation with *Pleurotus ostreatus* polysaccharides could decrease the relative abundance of *Proteobacteria*, which regulated the innate immunity on *Apostichopus japonicus* [28]. *G. lucidum* polysaccharide increased levels of short-chain fatty acids (SCFAs)-producing bacteria such as *Ruminococcus_1, Paraprevotella* and *Fusicatenibacter*, and decreased levels of *Escherichia-Shigella*, *Ruminococcaceae*, *Corynebacterium_1* and *Sutterella*, thereby reducing the animal disease activity index [29]. In conclusion, all these facts implied that mushroom polysaccharides could benefit the composition and metabolism of gut microbiota.

**Figure 1:**
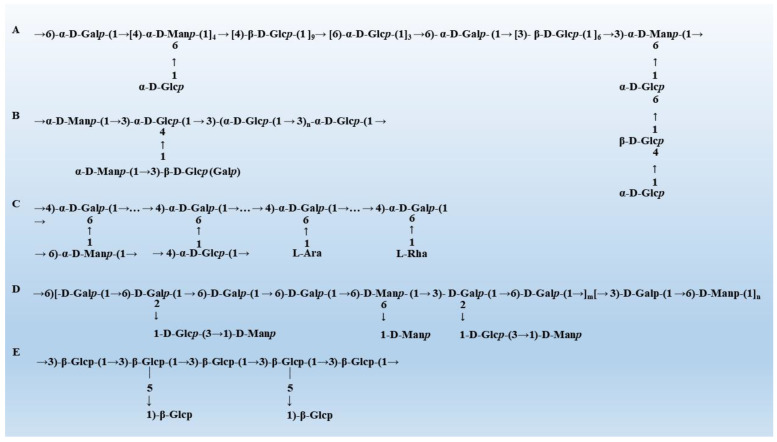
The typical structure of mushroom polysaccharides. (**A**) The structure of *Grifola frondosa* [31]. (**B**) The structure of *Hericium erinaceus* [32]. (**C**) The structure of *G. lucidum* [33]. (**D**) The structure of *Pleurotus eryngii* [34]. (**E**) The structure of *Lentinula edodes* [35].

The biological activity of mushroom polysaccharides is largely affected by its monosaccharide composition, molecular weight and the type of glycosidic bond [30]. Therefore, it is of great significance to explore the relationship between the structure of mushroom polysaccharides and the regulatory activity of gut microbiota for the treatment of diseases. Mushroom polysaccharides that have been reported usually exhibit the biological activity of specific structures. Due to the complex and diverse structure of polysaccharides, it is difficult to draw conclusions about the structure-activity relationship of polysaccharides from “special” to “universal”. This paper displays the typical structure of five mushroom polysaccharides (Figure 1) and summarizes the common structural characteristics of mushroom polysaccharides with gut microbiota regulation activity (Table 1).

### 2.1. Molecular Weight

The molecular weight is a key parameter for the activity of polysaccharides. The molecular weight of most polysaccharides was 10–800 kDa, but some polysaccharides had a molecular weight as high as 100,000 kDa or as low as 4 kDa. The molecular weight of *Ramaria flava* polysaccharide was 101.68 kDa, which could promote the production of acetic acid and propionic acid and stimulate the growth of *Lactobacillus rhamnosus* [38]. Tian et al. isolated three kinds of polysaccharides with different molecular weights from *H. erinaceus.* It was found that the polysaccharide with a molecular weight of 4 kDa was easily decomposed by gut microbiota, while the polysaccharide with a molecular weight of 823 kDa was hydrolyzed with difficultly [39]. Therefore, polysaccharides with a molecular weight that is either too large or too small are not conducive for the regulation of gut microbiota. However, there are exceptions to this inference. *Helvella leucopus* polysaccharide with an average molecular weight of 39.14 × 10^8^ Da could significantly improve the abundance of *Lactobacillus* and decrease the abundance of *Lachnospiraceae_NK4A136* and *Lachnospiraceae_unclassified* in DSS-induced colitis mice [40].

### 2.2. Monosaccharide Composition

On the basis of the monosaccharide composition, mushroom polysaccharides can be divided into glucan and heterosaccharide. Glucan is an isotype polysaccharide that is formed from glucose units by a glycosidic bond. It can be categorized as α- and β-glucan according to the type of glucoside bond [63]. Heterosaccharide is a polysaccharide composed of two or more different monosaccharides (glucose, galactose, mannose, xylose, etc.) [64]. Mushrooms rich in β-glucan have immunomodulatory activities [65]. Recent studies have shown that β-glucan could be fermented by the gut microbiome into SCFAs with immunomodulatory activity [66]. In addition, β-glucan can increase the number of beneficial bacteria such as *Bifidobacterium* and *Lactobacillus*, playing an important role in maintaining the balance of the gut microbiome [67]. Based on this conclusion, Saxami et al. emphasized that β-glucan-rich *P. eryngii* promoted the production of SCFAs and protected the integrity of the intestinal barrier [68]. The β-glucan in *L. edodes* has been shown to prevent cognitive impairment caused by a high-fat diet by improving the colon-brain axis [41]. For heterosaccharides, glucose, galactose and mannose are the most common monosaccharide residues, and they may play a pivotal role in the regulation of gut microbiota. Intestinal microorganisms can utilize *P. eryngii* polysaccharides that are rich in glucose (78.32%), galactose (8.47%) and mannose (9.43%) to produce acetic acid and propionic acid, while increasing the relative abundance of *Firmicutes* and decreasing the relative abundance of *Proteobacteria* and *Bacteroidetes* [43]. *H. erinaceus* polysaccharide, which consists of fructose, mannose, glucose and galactose, could increase the abundance of SCFAs-producing bacteria [39]. To date, no definitive conclusion has been drawn on the relationship between the regulation function of gut microbiota and monosaccharide composition. Notably, the activity of the regulating gut microbiota is higher when the monosaccharide composition of polysaccharides is more complex [69]. *F. velutipes* polysaccharide significantly promoted the proliferation of *Bifidobacteriaceae*, *Bacteroidaceae*, *Lachnospiraceae* and *Enterococcaceae*. However, *G. lucidum* polysaccharide only reduced the levels of *Oscillospira* and *Desulfovibrionaceae*, while *Poria cocos* sclerotium polysaccharide only increased the levels of *Lachnospiracea* and *Clostridium* [70,71]. The reason may be that *Flammulina velatus* polysaccharide is composed of glucose, mannose, xylose, fucose and galactose, while the monosaccharide of *G. lucidum* polysaccharide and *Poria cocos* sclerotium polysaccharide is mainly glucose.

### 2.3. Glycosidic Bonds

The relationship between the types and configurations of mushroom glucans glycosidic bonds and their immunomodulatory and antitumor activities has been proposed in many reports. The *G. frondosa* polysaccharides extracted by different methods had different structures, including α-1,6-, α-1,4-, β-1,6- and β-1, 3-glycosidic bonds, while (1→3, 1→6)-β-D-glucan is the main component with immunomodulatory and antitumor activities [72]. Both *Ganoderma sinense* glucan with main chains of (1→4)- and (1→6) -Glcp and *Ganoderma leucocontextum* glucan with main chains of →4)-α-D-Glcp-(1→4,6)-β-D-Glcp-(1→ have immunity-boosting effects [73,74]. *L. edodes* polysaccharide is a typical β-glucan with 1→3 link as the main chain and 1→6 link as the branch chain, which has significant immunomodulatory activity [75]. In addition, studies on the relationship between the types and configurations of heterosaccharides glycosidic bonds and the regulatory activity of gut microbiota have been reported. Xu et al. obtained a heteropolysaccharide (L2) with a molecular weight of 26 kDa from *L. edodes*, which was mainly composed of glucose and galactose, linked by 1→3 or 1→6 glycosidic bonds [36]. L2 could markedly increase the relative abundance of *Proteobacteria*, *Bacteroides acidifaciens*, *Alistipes* and *Helicobacter suncus* [27]. Furthermore, L2 could also reduce the abundance of age-related intestinal bacteria such as *Bacilli*, *Betaproteobacteria*, the *Firmicutes/Bacteroidetes* ratio and *Lactobacillaceae* [37]. The main chains of *Sparassis crispa* heteropolysaccharide (SCP-1) were (1→6)-α-D-Galp, (1→6)-β-D-Glcp and (1→3)-β-D-Glcp, and the side chains were (1→4)-β-D-Glcp, (1→3)-β-D-Glcp, T-α-L-Fucp and T-β-D-Glcp [45]. The SCP-1 has been shown to promote the production of SCFAs such as acetic acid, propionic acid and butyrate; elevate the levels of beneficial bacteria such as *Dialister* and *Megasphaera;* and inhibit the proliferation of harmful bacteria such as *Escherichia/Shigella* [44].

In conclusion, the complex structure of mushroom polysaccharides makes the relationship between mushroom polysaccharides and gut microbiota still in the preliminary stage. Although some studies have reported the relationship between the chemical structure of mushroom polysaccharides and the regulation of gut microbiota by mushroom polysaccharides, no general conclusions can be drawn. This paper suggests that the regulation effect of mushroom polysaccharides on gut microbiota is closely related to its molecular weight, monosaccharide composition and the type of glycosidic bond. It can be found that glucose, galactose, mannose and fucose were the monosaccharides that occurred frequently among the polysaccharides that possessed the regulatory function of gut microbiota, and the (1→3) and (1→6) linkage appeared frequently. *Firmicutes*, *Lactobacillus* and *Bacteroides* were the main regulated gut bacteria. However, the exact structure or the monosaccharides that stimulate specific gut bacteria have not yet been determined. Therefore, the relationship between them needs to be further studied.

## 3. Metabolism of Polysaccharides by Gut Microbiota

Polysaccharides are incapable of being decomposed and digested by saliva and under gastric and small intestinal conditions; hence, they are difficult to be absorbed by the body [76]. The reason is that only 17 polysaccharide digestive enzymes are encoded by the human genome; the remaining polysaccharide digestive enzymes are encoded by the microbes and their genomes in the human gut [77,78]. Polysaccharide digestive enzymes are responsible for the degradation and modification of polysaccharides, collectively known as carbohydrate-activated enzymes (CAZymes). According to the different catalytic mechanisms, CAZymes can be divided into six categories: glycoside hydrolases (GHs), polysaccharide lyases (PLs), carbohydrate esterases (CEs), glycosyltransferases (GTs), auxiliary activities (AAs) and carbohydrate-binding modules (CBMs) [79,80,81]. GHs and PLs are two types of enzymes that degrade glycosidic bonds, and contain 153 and 28 families, respectively. GHs breaks the glycosidic bonds between two or more carbohydrates and between carbohydrates and non-carbohydrates by inserting water molecules to degrade the main chain of carbohydrates. In contrast, PLs degrades the long chain of polysaccharides containing uronic acids through the β exclusion mechanism [82,83]. CEs remove polysaccharide ester groups and participate in carbohydrate side chain degradation [84]. CAZymes convert polysaccharides into a series of small-molecules that are easily absorbed, including SCFAs (acetic acid, propionic acid, butyric acid and iso-valerate acid), lipopolysaccharides and carbon monoxide [85]. The degradation of complex polysaccharides requires the cooperation of gut microbes, for example, the small molecule products formed by the degradation of inulin by *Bacteroidetes ovatus* can be further metabolized and utilized by *Bacteroidetes vulgatus* [86].

**Figure 2:**
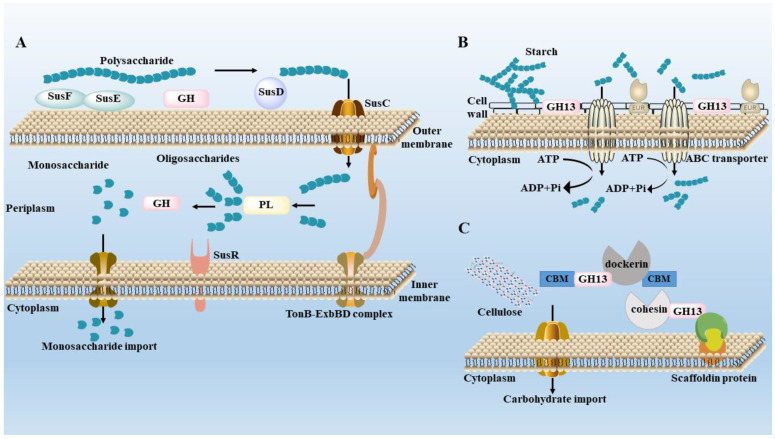
Degradation mechanisms of gut microbiota. (**A**) Starch utilization system (Sus) in *Bacteroides thetaiotaomicron*. (**B**) ATP-binding cassette (**A**–**C**) transport system in *Eubacterium rectale*. (**C**) The multi-enzyme complexes system in *Ruminococcus champanellensis*.

Figure 2 shows three polysaccharide degradation mechanisms of gut microbiota. The starch utilization system (Sus), ABC transport system and multienzyme complexes system are the main mechanisms for the degradation of polysaccharides by gut microbiota [87,88]. The Sus is an important way for *Bacteroides* to degrade polysaccharides. Sus R, as a transmembrane regulator, can detect the decomposition of polysaccharides. SusE and SusF proteins can recognize the polysaccharides and collect them at the cell surface. GHs decompose the polysaccharides into several oligosaccharides, which are bound by SusD protein and transported from the outer membrane into the periplasm by SusC protein. These oligosaccharides are further degraded into smaller oligosaccharides by GHs or PLs and further degraded into glucose by linking specific Sus A and Sus B before entering the cytoplasm [89]. Although *Firmicutes* have fewer genes encoding CAZymes, they encode more ABC transporters, phosphoenolpyruvate, carbohydrate PTS transporters, major facilitator superfamily transporters and glycoside-pentoside-hexuronide transporters to transport carbohydrates [90]. The multienzyme complexes system can efficiently degrade cellulose or resistant starch by interacting with dockerins and cohesins domains [91]. This system has been found in *Ruminococcus champanellensis* in human feces; dockerin is a small domain found in enzyme components and cohesin is a β-sandwich domain found in non-catalytic scaffold proteins that provides carbohydrate binding and/or cell wall anchoring functions [92]. These findings show that at least some *Firmicutes* in the microbiome have evolved to use cellulose in the human gut.

## 4. Effects of Mushroom Polysaccharides on Gut Microbe-Mediated Diseases

### 4.1. Improvement of Lipid and Glucose Metabolism Disorders

#### 4.1.1. T2DM

**Figure 3:**
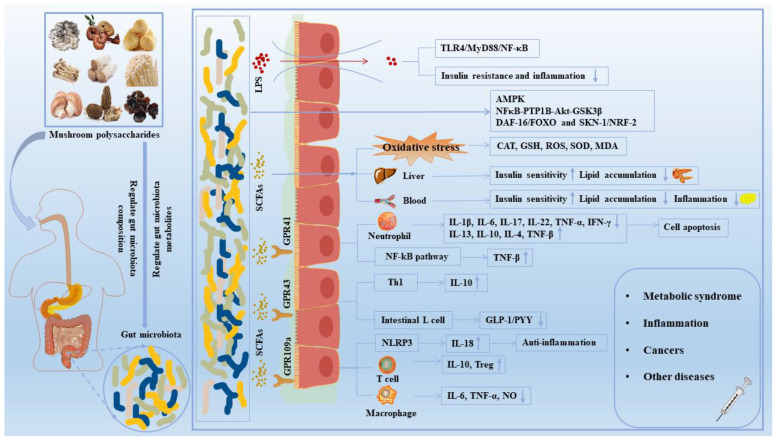
The effect of mushroom polysaccharides on human diseases by the regulation of gut microbiota. Mushroom polysaccharides could regulate the composition and relative abundance of gut microbiota and promote the production of SCFAs. SCFAs bond to receptors such as GPR41, GPR43 and GPR109A, activating downstream NF-κB, MAPK and other signaling pathways, thereby improving the intestinal mucosal barrier and maintaining intestinal homeostasis. In addition, SCFAs exerted an anti-diabetic and anti-obesity role by alleviating oxidative stress damage and regulating liver and blood biochemical indexes. Notably, harmful gut microbiota induced inflammation and insulin resistance by stimulating LPS secretion and activating TLR4/MyD88/NF-κB pathways. ↑, increased; ↓, decreased; CAT, catalase; GLP-1, glucagon-like peptide-1; GPR 109a, G protein-coupled receptor 109A; GPR 43, G protein-coupled receptor 43; GPR 41, G protein-coupled receptor 41; GSK3β, glycogen synthase kinase 3β; GSH, glutathione; IFN-γ, interferon-γ; IL, interleukin; LPS, lipopolysaccharide; MDA, malondialdehyde; MyD88, myeloid differentiation factor 88; NF-κB, nuclear factor-κB; NO, nitrogen monoxide; PYY, peptide YY; ROS, reactive oxygen species; SCFAs, short-chain fatty acids; SOD, superoxide dismutase; Th, helper T cell; TLR4, toll-like receptor 4; TNF-α, tumor necrosis factor-α.

Figure 3 shows the mechanism of mushroom polysaccharides on common diseases including T2DM. T2DM accounts for more than 95% of diabetes cases, mainly manifested as abnormal levels of glucose and lipids and low-intensity inflammation in both the blood and the liver [93]. The pathogeny of T2DM is due to dietary issues, physical inactivity, being overweight and being a smoker [94]. The common treatments for T2DM include insulin injections, oral sulfonylureas and biguanides drugs, and operative treatment [95]. However, these therapies may cause different degrees of side effects that greatly reduce the quality of life.

Mushroom polysaccharides could improve insulin resistance and promote gastrointestinal health by promoting the abundance of healthy bacteria and suppressing the abundance of harmful bacteria [96]. Table 2 summarizes the regulatory effects of typical mushroom polysaccharides on gut microbiota composition in chronic diseases, including T2DM. *Akkermansia muciniphila* accounts for 0.5–5% of the human intestinal tract, but it accounts for a lower proportion in obese and diabetic patients. This decrease results in damage to the intestinal barrier function and causes intestinal inflammation [97]. *G. frondosa* polysaccharides could reverse the level of *Akkermansia* in T2DM mice to inhibit the development of inflammation [60]. Moreover, *Armillariella tabescens* polysaccharides up-regulated the levels of *Lactobacillus* and *Ackermanella* to reduce lipopolysaccharide (LPS) content and improve intestinal barrier function, thereby reducing systemic inflammation in T2DM mice [98]. *G. lucidum* F31 increased the *Bacteroidetes*/*Firmicutes* ratio to improve glucose metabolism disorder and enriched SCFAs-producing bacteria including *Lactobacillus*, *Bacteroides* and *Ruminococcaceae* to improve the integrity of the intestinal barrier [99,100]. Thus, *G. lucidum* F31 could alleviate insulin resistance and inflammation by reducing the release of LPS from the gut into circulation [101]. Resistant starch encapsulated *G. lucidum* spores resulted in a synergistic hypoglycemic effect by enhancing glycolipid metabolism, insulin secretion and glycogen synthesis to reduce adipogenesis [102]. Moreover, *G. lucidum* polysaccharide-Chromium (III) complex (900 mg/kg day) supplements decreased the relative abundance of *Streptococcus*, *Enterococcus* and *Alistipes* and increased the relative abundance of *Enterorhabdus*, *Coriobacteriaceae* and *Micrococcaceae*, improving serum biochemical parameters, insulin sensitivity and glucose tolerance [103]. 

SCFAs were closely associated with the occurrence and development of T2DM and related metabolic diseases, which could protect the intestinal mucosal barrier, reduce inflammation levels and stimulate gastrointestinal movement [104]. SCFAs are mainly produced by specific gut microbiota through fermenting dietary fiber, including acetic acid, propionic acid, butyric acid and valeric acid [105]. The hypoglycemic mechanism of *Astragalus* polysaccharide was to decrease acetic acid and propionate concentration and increase butyric acid concentration [106]. *G. lucidum* sporoderm-broken spore polysaccharide increased the production of butyrate and acetate in high-fat-diet (HFD) mice [107]. The elevated intake of dietary fibers can increase the numbers of beneficial bacteria and then induce the production of SCFAs to activate G-protein coupled receptor (GPR43), comprehensively improving the blood glucose homeostasis of patients with T2DM [108]. *Fomitopsis castaneus Imaz* exopolysaccharides increased the contents of SCFAs in the digestive system of children, especially for butyric acid. 

Impaired glucose tolerance and impaired fasting glucose referred to an intermediate transition state between the normal condition and diabetes, with a high risk of T2DM. *Phellinus linteus* polysaccharides significantly reduced fasting blood glucose levels and improved oral glucose tolerance by increasing the ratio of phosphatidylcholine to phosphatidyl ethanolamine and the ratio of S-adenosylmethionine to S-adenosylhomocysteine [109]. Oral *G. frondosa* polysaccharides reduced serum fasting blood glucose, oral glucose tolerance, cholesterol, triglyceride, and low-density lipoprotein cholesterol levels, as well as levels of cholesterol, triglyceride, and free fatty acids in the liver. The relative abundance of *Streptococcus*, *Enterococcus*, *Staphylococcus* and *Pneumococcus* decreased with high concentration of *G. frondosa* polysaccharides [20]. These results indicated that *Streptococcus*, *Enterococcus*, *Staphylococcus,* and *Pneumococcus* were positively correlated with serum and liver lipid biochemical parameters, further proving a close relationship between metabolic parameters and intestinal microbiota. *Auricularia auricula* polysaccharides alleviated insulin resistance and improved blood glucose stability through restoring amino acid metabolism, lipid metabolism, bile acid synthesis and glycerophospholipid pathways [110]. 

Many studies revealed that mushroom polysaccharides regulated glucose and lipid metabolism by activating related protein expression in metabolic pathways. *Auricularia auricula-judae* polysaccharides improved glucose-lipid metabolism disorders by activating protein kinase B (AKT) and adenosine monophosphate-activated protein kinase (AMPK) signaling pathways in T2DM mice [111]. Using 55% ethanol extraction for *G. frondose* inhibited the liver stearoyl-Coenzyme A (CoA) desaturase 1, sterol regulatory element-binding transcription factor-1c and acetyl CoA carboxylase signaling pathways by increasing the levels of AKT1, glucokinase, AMPK-α and cholesterol 7-α hydroxylase, thereby improving glucose and lipid metabolism disorders [112]. Therefore, regulation of specific signaling pathways could reverse blood glucose, blood lipids and the liver index as well as improve the gut microbiota, thus resolving metabolic disorders of the liver.

**Table 2:** The modulation of gut microbiota in metabolic disorders by mushroom polysaccharides.

Disease	Mushroom	Model	Gut Microbiota Regulation	Effects on Hosts & Functional Mechanisms	Ref.
T2DM	*Grifola* *frondosa*	STZ-induced KM mice	*Alistipes*↑ *Streptococcus*, *Enterococcus*, *Staphylococcus* and *Aerococcus*↓	Reduced the serum levels of FBG, OGT, TC, TG and LDL-C, and decreased the hepatic levels of TC, TG and FFA; increased mRNA expression of CYP7A1 and BSEP.	[20]
T2DM	*Ganoderma* *lucidum*	STZ-induced mice	*Blautia*, *Dehalobacterium*, *Parabacteroides* and *Bacteroide*s↑ *Aerococcus*, *Ruminococcus*, *Corynebactrium* and *Proteus*↓	Decreased the levels of fasting blood glucose and insulin; restored the amino acids metabolism, carbohydrates metabolism, inflammatory substances metabolism.	[51]
Hyperlipidemia	*Grifola* *frondosa*	HFD-induced Wistar rats	*Helicobater*, *Intestinimonas*, *Parasutterella*, *Ruminococcus* and *Flavonifracter*↑ *Clostridium-XVIII*, *Butyricicoccus* and *Turicibacter*↓	Through decreasing the serum TG, TC, and FFA levels, and increasing the serum HDL-C level; increased the mRNA levels of BSEP, CYP7A1, Acox1 and hepatic GS.	[59]
T2DM	*Grifola* *frondosa*	STZ-induced ICR mice	*Porphyromonas gingivalis*, *Akkermansia muciniphila*, *Lactobacillus acidophilus*, *Bacteroides acidifaciens*↑ *Firmicutes/Bacteroidetes ratio* and *Proteobacteria*↓	Decreased the fasting blood glucose level, improved oral glucose tolerance, alleviated insulin resistance; activated IRS1, PI3K, and GLUT4, inhibited JNK1/2; regulated the IRS1/PI3K and JNK signaling.	[60]
T2DM	*Ganoderma* *lucidum*	HFD-induced and STZ-induced KM mice	*Ruminococcaceae*, *Prevotellaceae* and *Peptococcaceae*↑ *Lachnospiraceae*, *Desulfovibrionaceae* and *Lactobacillaceae*↓	Repaired islet cells and increased insulin secretion, improved insulin resistance, and improved carbohydrate metabolism, amino acid metabolism and lipid metabolism.	[101]
T2DM	*Ganoderma* *lucidum*	STZ-induced SD rats	*Lactobacillus*↑ *Proteobacteria*↓	Promoted the expression of GS2, GYG1, Insig1, Insig2, ACC; elevated the level of HDL-C and reduced levels of TC and TG.	[102]
T2DM	*Grifola* *frondosa*	STZ-induced KM mice	*Alistipes*↑ *Streptococcus*, *Enterococcus*, *Staphylococcus* and *Aerococcus*↓	Improved abnormal serum biochemical indicators TG, TC, LDL-C and glucose, inhibited lipid accumulation and steatosis; downregulated CD36 and SREBP-1C, upregulated CYP7A1.	[103]
Obesity	*Ganoderma* *lucidum*	HFD-induced C57BL/6J mice	*Akkermansia*, *Bifidobacterium*, *Turicibacter*, *Parabacteroides*↑ *Blautia*, *Rikenella*, *Ruminiclostridium_UGC-009* and *Lachnospiraceae*↓	Inhibited fat accumulation and body weight, hyperlipidemia; reduced LPS level; decreased levels of TNF-α and IL-1β; increased acetate and butyrate production; inhibited LPS/TLR4/NF-κB signaling pathway.	[107]
T2DM	*Grifola* *frondosa*	STZ-induced ICR mice	*Lactobacillus*, *Desulfovibrio*, *Helicobacter*, *Lactobacillus* and *Bacteroides*↑ *Verrucomicrobia*, *Ruminococcus* and *Prevotella*↓	Improved the composition of gut microbiota and promoted the proliferation of beneficial bacteria.	[113]
Obesity	Agrocybe cylindracea	HFD-induced C57BL/6J mice	*Bacteroides*, *Parabacteroides*, *Butyricimonas* and *Dubosiella*↑ *Desulfovibrio* and *Oscillibacter*↓	Reduced the levels of obesity-related TNF-α and IL-6, reduced fasting glucose and insulin levels.	[114]
T2DM	*Sanghuang-* *porous* *vaninii*	high-fat and high-sucrose ICR mice	*Akkermansia*, *Dubosiella*, *Bacteroides* and *Parabacteroides*↑ *Lactobacillus*, *Flavonifractor*, *Odoribacter* and *Desulfovibrio*↓	Improved body weight, glycolipid metabolism, and inflammation-related parameters; ameliorated pancreas and jejunum injuries; enriched insulin signaling pathway and PI3K-Akt signaling pathway.	[115]
Obesity	*Grifola* *frondosa*	HFD-induced C57BL/6JNju mice	*Mucispirillum*, *Bilophila* and *Dehalobacterium*, *Sutterella*↑ *Coprococcus* and *Ruminococcus*↓	Controlled the body weight, blood glucose and related organ indices, counteracted hyperlipidemia and IR triggered; regulated AST and ALT; down-regulated TLR4/NF-κB signaling.	[116]
NAFLD	*Lentinan*	HFD-induced C57BL/6J mice	*Bifidobacterium*, *Streptococcaceae* and *Enterococcaceae* genus, *Streptococcus*, *Enterococcus*, *Ruminococcaceae*↑ *Helicobacteraceae* and *Helicobacter*↓	Restored intestinal redox balance, and reduced serum LPS; altered inflammation-insulin (NFκB-PTP1B- Akt-GSK3β) signaling molecules.	[117]
NAFLD	*Grifola* *frondosa*	HFD-induced Wistar rats	*Bacteroides*, *Bifidobacterium*, *Blautia*, *Coprococcus*, *Phascolarctobacterium*, *Prevotella*↑ *Alistipes*, *Flavonifractor*, *Paraprevotella* and *Oscillibacter*↓	Modulated the expression of specific gene related to lipid synthesis and conversion, CYP4A1, ACC, TNF-α, SOCS2 and CYP7A1; reduced hepatocyte steatosis and liver cell injury.	[118]
Hyperlipidemia	*Auricularia auricular*	HFD-induced SD rats	*Firmicutes*, *Roseburia*, *Flavonifractor* and *Clostridium IV*↑ *Bacteroidetes*↓	Reduced the levels of TC and LDL-C; induced the significant growth of SCFA-producing bacteria and the accumulation of SCFAs concentrations.	[119]
Obesity	*Pleurotus* *eryngiion*	HFD-induced C57BL/6J mice	*Lactococcus*↑ *Roseburia*↓	Suppressed fat accumulation; decreased LDL-C; increased fecal bile acids; increased the concentration of SCFAs.	[120]
T2DM	*G. frondosa*	HFD-induced SD rats	*Bacteroidetes/Firmicutes*, *Lactobacillus* and *Turicibacter*↑ *Prevotella* and *Bifidobacterium*↓	Decreased the expression levels of TNF-α, IL-1β and IL-6; alleviated inflammation by the TLR4/MyD88/NF-κB pathway.	[121]
Obesity	*Ganoderma* *lucidum*	HFD-induced C57BL/6 mice	*Bacteroides* spp., *Anaerotruncus colihominis*, *Clostridium*↑ *Enterococcus* spp., *Lactococcus lactis* and *Oscillibacter valericigenes*↓	Improved gut barrier integrity, reduced endotoxemia, decreased TLR4 signal and inflammation; reduced the number of macrophages.	[122]
Hypercholesterolemia	*Ganoderma* *lucidum*	HCD-induced C57BL/6 mice	*Faecalibacterium prausnitzii*, *Lactobacillus* and *Prevotella*↑ *Bacteroides acidifaciens*, *Mucispirillum schaedleri* and *Parabacteroides distasonis*↓	Prevented FA synthesis and accumulation through down-modulating genes involved in lipogenesis, elongation and desaturation; activation of PPARs, fatty acid oxidation and bile acid conversion.	[123]
Hyperlipidemia	*Ganoderma* *lucidum*	HFD-induced Syrian golden hamsters	*Ruminococcus*, *Oscillibacter*, *Bifidobacterium*, *Prevotella* and *Alistipes*↑ *Desulfovibrio*, *Clostridium*↓	Alleviated the serum levels of TG, TC, and LDL-C; decreased the serum levels of AST; increased beneficial bacteria and reduced harmful bacteria.	[124]

Note: ↑, increased; ↓, decreased; ACC: acetyl CoA carboxylase; Acox1: acyl-Coenzyme A oxidase 1; AKT, protein kinase B; ALT: alpha-alanine aminotransferase; AST: aspartate aminotransferase; BSEP: bile salt export pump; CYP4A1: cholesterol 4 alpha-hydroxylase; CYP7A1: cholesterol 7 alpha-hydroxylase; FBG: fasting blood glucose; FFA: free fatty acids; GLUT4: glucose transporter 4; GSK3β, glycogen synthase kinase 3β; GS2: glycogen synthase 2; GYG1: glycogenin-1; HDL-C: high-density lipoprotein cholesterol; HFD: high-fat diet; IL-6: interleukin-6; IL-1β: interleukin-1β; IR: insulin resistance; IRS1: insulin receptor substrate 1; JNK, c-Jun N-terminal kinase; KM, kunming; LDL-C: low-density lipoprotein cholesterol; LPS: lipopolysaccharide; MyD88: myeloid differentiation factor 88; NF-κB: nuclear factor-κB; OGT: oral glucose tolerance; PI3K: phosphatidylinositol 3 kinase; PPAR: peroxisome proliferator-activated receptors; Ref: reference; SCFA: short-chain fatty acid; SOCS2: suppressors of cytokine signaling 2; SREBP-1c: sterol regulatory element-binding protein-1c; STZ: streptozocin; T2DM, type 2 diabetic mellitus; TC: total cholesterol; TG: triglyceride; TLR4: toll-like receptor 4; TNF-α: tumor necrosis factor-α.

#### 4.1.2. Obesity

Obesity is a serious public health issue affecting everyone, characterized by metabolic disorders accompanied by changes in gut microbiome composition and diversity [125]. It is defined as abnormal or excessive fat accumulation, mainly caused by a sedentary lifestyle and an imbalance between energy intake and consumption. Being obese or overweight is associated with an increased risk of many chronic diseases such as T2DM, hypertension, cardiovascular disease and hyperlipidemia, so it is crucial to elucidate how to prevent obesity in modern medical research [126]. Notably, improving diet and lifestyle or pharmacotherapy may be the options for treating obesity, but these methods may not have the desired anti-obese effect. Surgical treatment still has certain limitations and risks [127]. 

Changes in gut microbiota composition were closely associated with the development of obesity and related metabolic diseases [128]. A large number of clinical and experimental studies showed that gut microbiota plays a key role in the occurrence and development of obesity by regulating the host’s energy metabolism, substrate metabolism and inflammatory response. The content of LPS-producing *Proteobacteria* and the proportions of *Firmicutes* and *Bacteroidetes* in HFD-induced mice were significantly higher than those of normal-fed mice [129]. Among them, *Bacteroidetes* and *Firmicutes* have a mutually reinforcing symbiotic relationship that can jointly promote host intestinal energy absorption or storage. Thus, gut microbiota is a promising strategy for improving obesity and related metabolic disorders.

Polysaccharides could reduce weight by inhibiting the growth of obese gut microbiome and increasing the level of microbiota-derived metabolites [130]. The effects of *Agrocybe cylindracea* polysaccharide on adipose accumulation and weight loss in HFD-induced mice have been reported, and it was found that *Desulfovibrio* was decreased and *Parabacteroides* was increased, which markedly reduced the levels of obesity-related tumor necrosis factor α (TNF-α) and interleukin-6 (IL-6) [114]. *Sanghuangporous vaninii* ethanol extract observably improved body weight and glucose and lipid metabolism by elevating the relative abundance of *Akkermansia*, *Dubosiella*, *Bacteroides* and *Parabacteroides* and reducing the relative abundance of *Lactobacillus, Flavonifractor*, *Odoribacter* and *Desulfovibrio*. Furthermore, it enriched glycerolipid metabolism, insulin signaling pathway and fatty acid degradation in T2DM-induced mice [115]. *G. lucidum* sporoderm-broken spore polysaccharides significantly inhibited weight in HFD mice after treatment for 9 weeks. Its underlying mechanisms were increased levels of SCFAs, improved intestinal barrier function, reduced endotoxemia and increased production of beneficial bacteria. The regulation of gut microbiota was mainly manifested by *G. lucidum* sporoderm-broken spore polysaccharide that reversed the relative abundance of many bacteria in HFD-fed mice, particularly some potential probiotics including *Allobaculum* and *Bifidobacterium*, which showed a positive correlation with anti-obesity [107]. *Bifidobacterium*, *Lactobacillus* and *Akkermansia* could promote the production of SCFAs and inhibit the abundance of *Clostridiaceae*, *Desulfovibrio* and *Enterococcus*, which would help reduce body weight and lipid accumulation [131]. Butyric acid in SCFAs may alleviate diet-induced obesity, and another study showed that adding butyric acid to the diet can prevent diet-induced obesity by promoting the transition from adipogenesis to lipid oxidation [132]. Inconsistently, one study showed that butyric acid can promote energy absorption and thus contribute to the development of obesity [133]. 

*Ganoderma applanatum* polysaccharides significantly reduced total cholesterol, triglycerides, low-density lipoprotein cholesterol levels and atherosclerotic index in obese rats, improving the symptoms of dyslipidemia [134]. The water-soluble polysaccharide components of *P. eryngii* reduced adiposity and cholesterol via inhibiting the intestinal circulation of bile acids and up-regulating the mRNA levels of sterol-regulatory element binding proteins 2 and its target gene low-density lipoprotein receptor in the liver [135]. Both *G. lucidum* polysaccharide and *G. frondosa* polysaccharides could significantly improve blood glucose and organ indexes of HFD mice and reverse the changes in gut microbiota caused by obesity. *G. frondosa* polysaccharides promoted lipid metabolism and homeostasis by activating Decay Accelerating Factor16/Forkhead Box O and SKN-1/nuclear factor erythroid 2-related factor 2 (Nrf2) signal pathways or inhibiting Toll-like receptor 4 (TLR4)/NF-κB signal transduction, all of which are associated with inflammation and insulin resistance [116,136]. *G. lucidum* polysaccharides inhibited HFD-induced splenic lymphocyte apoptosis by reducing the ratio of Bax (Bcl2-associated X protein)/Bcl-2 (B-cell lymphoma-2) and inhibiting the activation of caspase-3 [19]. *Astragalus* polysaccharides regulated Cux1 by Mir-1258-5p to promote the differentiation of C3H10T 1/2 cells into brown adipocytes, increasing energy consumption; this may be used as a treatment for obesity [137]. *Volvariella volvacea* polysaccharides reduced fat accumulation by regulating the AAK-2/NHR-49 mediated fatty acid synthase pathway and the ACS-2 mediated fatty acid oxidation pathway [138].

#### 4.1.3. NAFLD

NAFLD is the most common chronic liver disease and is generally considered a common metabolic disorder closely related to obesity. Previous research even indicated that overweight or obese subjects lost at least 5% of their body weight was the only effective strategy for treating NAFLD and non-alcoholic steatohepatitis [139]. The pathogenesis of NAFLD was described as a “two-hit” hypothesis. Firstly, insulin resistance promotes excessive hepatic fat accumulation, leading to liver sensitivity. The secondary pathogenic damage of oxidative stress is that it induces inflammation and cell death that are also caused by gut microbiota changes [140]. An imbalance in the relative abundance of *Bacteroidetes* and *Firmicutes* has been found in NAFLD patients, which leads to an increase in microbial metabolites such as SCFAs, lipopolysaccharides and secondary bile acids that can cross the intestinal barrier and reach the liver, contributing to inflammation and disease progression [141,142].

Mushrooms could promote intestinal health by decreasing intestinal lipid uptake and influencing intestinal microbiota. *Agaricus bisporus* mushroom positively affected human intestinal health by increasing the abundance of *Bacteroidetes* and decreasing the abundance of *Firmicutes* [143]. *Lentinan* increased levels of phylum *Actinobacteria* and decreased the levels of phylum *Proteobacteria* and *Epsilonbacteraeota,* alleviating intestinal microbiota disorder in HFD mice. Moreover, it also improved NFκB-PTP1B-Akt-GSK3β (inflammation-insulin) signaling pathways to prevent hepatic steatohepatitis [117]. *G. frondosa* polysaccharide significantly increased the composition of beneficial bacteria, especially *Alistipes*, *Flavonifractor* and *Oscillibacter,* which may play an important role in preventing NAFLD [118]. In addition to the above-mentioned edible fungi, the H1 component of the *Hirsutella sinensis* polysaccharide reversed the dysbacteriosis caused by HFD, and promoted the number of neomycin-sensitive bacteria such as *Parabacteroides. goldsteinii mycelium*, which indirectly improved glycolipid metabolism disorders [144]. Cauliflower mushroom extract combined with the fermented fungus JS can alleviate the lipid metabolism disorders of rats treated with ovarian resection; the changes of intestinal flora and menopausal symptoms caused by estrogen deficiency, such as tail skin temperature increase, visceral fat volume increase, dyslipidemia and glucose intolerance, all of which were similar effects compared with positive drugs [145].

Mitochondria play a central role in the progression of NAFLD and mainly control cell death signaling. A chronic imbalance between lipid anabolism and catabolic processes in the liver leads to mitochondrial dysfunction [146]. Mushroom ingestion may alleviate the metabolic burden of liver mitochondria through reducing insulin resistance and hepatic steatosis. *Bletilla striata* polysaccharide showed great potential in treating NAFLD, which markedly regulated the liver metabolism of fatty acids, arachidonic acid and other related metabolites in HFD-fed mice, and reduced lipid accumulation and fibrosis in liver tissues [147]. *Antrodia cinnamomea* alleviated oxidative stress and inflammation to inhibit fat production by up-regulating aldehyde dehydrogenase 2 activity and accelerating the elimination of reactive oxygen species [148]. 

### 4.2. Immunoregulation Effects

Changes in gut microbiota are closely related to the immune system and other inflammatory conditions. There is a growing awareness that the microbiome is crucial for regulating the immune system. IBD is a chronic, multifactorial, high-incidence gastrointestinal inflammatory disease that includes ulcerative colitis and Crohn’s disease [149]. The etiology of IBD may be microbial symbiosis and immunity, intestinal barrier rupture, oxidative stress and DNA damage [150,151]. Various factors such as dietary patterns, environmental changes, genetic predispositions and adaptive immune responses can contribute to IBD [152]. A high-fat diet or lack of dietary polysaccharides in the diet promoted an imbalanced gut microbiome, which degraded host mucosal polysaccharides, destroyed the intestinal mucosal barrier and increased the secretion of LPS. LPS can enter the circulatory system through the mesenteric veins and act on target organs and tissues, causing intestinal inflammation or inducing colorectal cancer [153]. Current pharmacologic treatments for IBD mainly include aminosalicylates, glucocorticoids, immunosuppressants, biologics and novel small molecule drugs, but they all have certain limitations in terms of efficacy and safety [154]. Thus, treatment of IBD with safe, low-toxicant and efficient mushroom polysaccharides has been widely explored.

Table 3 exhibits the regulatory effects of typical mushroom polysaccharides on gut microbiota composition in IBD and other inflammatory models. Previous studies found that *Lactobacillus* spp. could alleviate DSS-induced colitis by modulating gut microbiota composition and stimulating natural killer (NK) cells, macrophages and T lymphocytes [155]. *Bifidobacterium breve* relieved DSS-induced colitis by reducing the levels of TNF-α, interleukin-1β (IL-1β), IL-6 and partially restoring the unbalanced gut microbiome [156]. Other studies also verified that anti-inflammatory microorganisms such as *Lactobacillus* spp., *Bacteroides*, *Bifidobacterium* and *Prevoella* were significantly reduced in trinitro-benzene-sulfonic acid-induced rat colon, while pro-inflammatory bacteria such as *Corynebacteriums*, *Staphylococcus* and *Rumencoccus* were enriched [157]. However, a water-soluble *G. lucidum* mycelium extract could reverse this gut microbiota imbalance [158]. Some other studies also indicated that *G. lucidum* polysaccharides improved the composition of intestinal microbiota in DSS-induced mice by reducing the abundance of *Escherichia/Shigella*, *Enterococcus* and *Staphylococcus* [159].

Different mushroom polysaccharides showed different immunomodulatory pathways in IBD. *G. lucidum* polysaccharides attenuated inflammatory factors in LPS-activated macrophages in mice; this anti-inflammatory effect was mediated by inhibiting NF-κB and MAPK signaling pathways [167]. Another study used *Lentinan*-loaded Budesonide (LNT/BuD-NPS) to treat ulcerative colitis and significantly alleviated inflammation by inhibiting the TLR4/MyD88/NF-κB signaling pathway [168]. SCFAs possessed the function of improving the intestinal mucosal barrier and stimulating the production of immunosuppressive cytokines [152]. The immunomodulatory effects should be attributed to SCFAs, which acted as signaling molecules to regulate and maintain the host’s immune system [169]. *Bacteroides* spp. fermented polysaccharides could produce SCFAs such as acetic acid, propionic acid and butyric acid that inhibited the production of pro-inflammatory cytokines, enhanced the expression of interleukin-10 (IL-10) and activated Treg cells, all of which were important in improving chronic inflammatory diseases and promoting colon cell health [170]. Acetic acid and butyric acid in SCFAs can activate GPR41 and GPR43 and inhibit histone deacetylase activity to exert anti-inflammatory effects [171].

In addition to intestinal inflammation, other inflammatory diseases also affect the microbiome homeostasis in the intestinal tract. Increasing the activity of antioxidant enzymes can protect oxidative damage to the pancreas caused by the proliferation of *Helicobacter pylori* [172]. Thus, increased oxidative stress was thought to be the pathogenesis of pancreatitis. Selenium-lentinan intake could enhance the activity of superoxide dismutase and glutathione peroxidase to alleviate oxidative stress [173]. *G. lucidum* strain polysaccharides alleviated oxidative stress by inhibiting the overgrowth of *Bacteroides*, *Prevotalles* and *Helicobacter* in mice induced by diethyldithiocarbamate [160]. *Bacteroides* were negatively correlated with lipase and trypsin, while *Firmicutes* were negatively correlated with glutathione peroxidase. Inonotus obliquus polysaccharides increased the relative abundance of *Bacteroides* and decreased the abundance of *Firmicutes* in pancreatitis mice, thereby reducing levels of serum pro-inflammatory cytokines [25].

Polysaccharides affect the number of intestinal immune cells by regulating the composition of gut microbiota, and then affect the secretion of cytokines such as immunoglobulin interleukin-1α (IL-1α) and interleukin-2 (IL-2), which play a crucial role in the mucosal immune system. *G. lucidum* polysaccharides significantly reduced the proportion of *Firmicuteum* and *Bacteroidetes*, and increased the levels of immune interferon-γ (IFN-γ), IL-2, interleukin-4 (IL-4) and other serum cytokines that strengthen the intestinal barrier [174]. Lentinan can increase the diversity of gut microbiota; reduce the ratio between *Firmicutes* and *Bacteroidetes;* promote the proliferation of splenic lymphocytes in vitro; increase the levels of TNF-α, IL-1α and IL-2; and enhance the immunity of elderly mice [37]. *Poria cocos* polysaccharides reduced chronic prostatitis by regulating the levels of anti-inflammatory and pro-inflammatory factors [175].

### 4.3. Antitumor Effects

Appropriate regulation of the immune response could reduce the risk of pathogen invasion caused by inflammatory responses and thus maintain a healthy gastrointestinal system [176]. However, excessive immune regulation can disrupt intestinal homeostasis and promote the metastasis of normal cells to malignant cells [177]. Since gut microbiota is closely associated with the inflammatory response, gut microbiota may also be indirectly involved in carcinogenesis by regulating local and systemic immune responses. Currently, mainstream cancer therapeutic approaches include chemotherapy, radiation therapy and surgery [178]. These traditional treatments cause side effects, but their safety and efficacy are still questionable. 

Changes in gut microbiota have been reported to affect the incidence of cancer [179]. Some gut microbiota can also be used as predictive biomarkers for the early detection of cancers. Many well-defined species that promoted the development of colorectal cancer include *Fusobacterium* spp., *Streptococcus bovis*, *Bacteroides fragilis*, *superoxide-producing Enterococcus*. *faecalis*, *Streptococcus gallolyticus*, *Peptostreptococcus* spp., and *Porphyromonas* spp. [180]. *Helicobacter pylori* has been identified as a Class I carcinogen for stomach cancer [181]. *Enterobacteriaceae* was associated with human colon carcinogenesis [182]. Ingestion of a probiotic *Lactobacillus Scidophilus* could inhibit mouse tumor growth [183]. *Bifidobacteria* has beneficial effects on the host and improves tumor-specific immunity by enhancing dendritic cell functions [184]. 

In recent years, increasing research has focused on the antitumor effect of mushroom polysaccharides. However, only a few studies have focused on the interaction between polysaccharides and cancer based on gut microbiota which mainly reported the treatment of breast cancer and colon cancer with *G. lucidum* polysaccharides [185]. *G. lucidum* spore extract (ESG) reshaped the intestinal microbiota in 4T1 tumor-bearing mice: there was an increase in the relative abundance of *Firmicutes* and *Proteobacteria* and a decrease in the relative abundance of *Actinobacteria*, *Bacteroidetes* and *Cyanobacteria* [186]. In the same year, another study showed similar conclusions that *G. lucidum* polysaccharides combined with paclitaxel exerted an antitumor effect on 4T1 breast tumor-bearing mice. The combined treatment significantly enriched five genera such as *Bacteroidetes* and *Ruminococcus* and reduced the abundance of *Desulfovibrios* and *Odoribacter*, which balanced intestinal flora and inhibited tumor metabolism [187]. *G. lucidum* polysaccharides regulated the relative abundance of beneficial bacteria such as *Lactobacillus* and *Bifidobacterium* to induce the production of SCFAs, improving intestinal barrier damage and inhibiting the TLR4/MyD88/NF-κB signal pathway, thereby reducing the risk of colitis and carcinogenesis [161]. Gynostemma pentaphyllum combined with *G. lucidum* polysaccharides markedly promoted the abundance of SCFAs-producing bacteria, elevated butyrate and iso-butyrate levels and suppressed the abundance of sulfate-reducing bacteria [188]. The abundance of *Oscillospira* was higher in colorectal cancer mice, *G. lucidum* polysaccharides could reverse the abundance of this bacterium and also reduce the number of *Desulfovibrionaceae*. In addition, four cancer-related genes including Acaa1b, Fabp4, Mgll and stearoyl-CoA desaturase 1 were down-regulated [70]. Thus, reducing specific bacteria, rather than ensuring recovery of the overall microbial diversity and regulation of cancer-associated genes, was the mainspring for alleviating colorectal cancer. *Carboxymethylated Poria* alleviated colon injury induced by 5-fluorouracil in CT26 tumor-bearing mice. The diversity of gut microbiota was restored by increasing the proportion of *Bacteroidetes*, *Lactobacillus* and butyric acid- and acetic acid-producing bacteria. In addition, *Carboxymethylated Poria* also increased the levels of Nrf2 and Bcl-2 and decreased the levels of NF-κB, P-P38 and Bax [189]. In conclusion, mushroom polysaccharides remodeled the gut microbiota and inhibited tumor growth, possibly due to the secondary metabolites produced by gut microbiota selectively fermented mushroom polysaccharides. 

SCFAs are important signaling molecules and the end-products of metabolic reactions that could reduce the risk of colorectal cancer by regulating the immune system and improving the integrity of the intestinal barrier [190]. Targeted changes in the proportion of SCFAs will affect the relationship between gut microbiota and host health [191]. Degradation of macromolecular carbohydrates to produce SCFAs is one of the main mechanisms of the gut microbiota for inhibiting tumor growth [192]. Indigestible mushroom polysaccharides that are fermented by gut microbiota could produce SCFAs and further regulate the intestinal microenvironment. It had been reported that propionate and n-butyrate possessed anti-tumor properties [193]. *F. velutipes* polysaccharides increased the content of bacterial metabolites such as acetic acid, butyric acid and propionic acid that promoted the development and maintenance of the immune system, thus reducing intestinal inflammation and the occurrence of tumors [26]. One study suggested the antitumor effects of polysaccharides from *G. lucidum* and *G. Sinense*, which had similar effects and mechanisms in inducing macrophage phagocytosis, producing cytokines and inhibiting the activity and migration of breast cancer cells. Their anti-tumor activity was verified by enriching *Alistipes* that produced SCFAs, which activated the TLR4-related MAPK/NF-κB signaling pathway [194]. However, the anti-tumor mechanisms of polysaccharides still need further research. 

### 4.4. Other Beneficial Effects

With increasing research on the gut microbiota, dysregulation of the gut microbiota affected not only intestinal diseases, metabolic diseases and cancers, but also induced psychiatric disorders such as Parkinson’s disease (PD) and Alzheimer’s disease (AD) [195]. Dysregulation of the gut microbiome could increase intestinal permeability and systemic inflammation, which may induce learning, memory and other cognitive impairments through the “microbio-gut-brain” axis [196]. A recent study found significant differences in the abundance of gut microbiota in AD patients at the phylum level compared to those with healthy gut microbiota, with a decrease in *Firmicutes* and an increase in *Bacteroides* [197]. Zhang et al. showed that *Sparassis crispa* polysaccharide may play an anti-AD role by reshaping the gut microbiota composition and inhibiting inflammation. In terms of regulating the gut microbiota, the relative abundance of *ventriosum group*, *Lachnospiraceae_UCG_010*, and *Lachnospiraceae_UCG_001* was increased, and the growth of *Escherichia/Shigella* was inhibited [198]. Gao et al. proposed that *Cistanche deserticola* polysaccharide improved cognitive function in D-galactose-induced aging mouse models by restoring gut microbial homeostasis, thereby reducing oxidative stress and peripheral inflammation [199]. Another study showed that a galactoglucan isolated from *Cistanche deserticola* could promote the growth of *Bacteroides* and *Lactobacillus* [200]. *F. velutipes* polysaccharides enhanced scopolamine-induced learning and memory impairment by mediating intestinal flora and inhibiting inflammation. The results showed that *F. velutipes* polysaccharides-fed mice significantly increased the number of platform crossings and swimming distances in the probe test. The relative abundance of *Bacteroidia*, *Erysipelotrichia* and *Actinobacteria* were increased, while the relative abundance of *Clostridia* and *Bacilli* were decreased. Inflammatory factors such as IL-1β, TNF-α, IL-6 and IL-10 were also suppressed [79]. The SCFAs produced by the degradation of polysaccharides by the gut microbiota can not only maintain the intestinal barrier function and intestinal homeostasis, but also directly or indirectly affect brain function. Studies have shown that butyrate intake can regulate cytoneurotrophic factors, thereby improving cognitive function and having neuroprotective effects [201]. Additionally, existing research has found that mushroom polysaccharides such as *Amanita caesarea* polysaccharide, *Hericium erinaceus* polysaccharide and *Armillaria mellea* polysaccharide could reduce AD through an antioxidant effect, which is mainly manifested as enhancing the Nrf2 cascade reaction; increasing the contents of endogenous antioxidant enzymes GSH-Px, CAT and SOD; and reducing oxidation indexes such as MDA and 4-Hydroxynonenal [202,203,204]. 

PD is the second most serious neurodegenerative disease affecting the elderly, resulting in symptoms such as salivation, difficulty in swallowing and delayed gastric emptying that seriously affect patients’ quality of life. The unique changes in the gut microbiota of PD patients can be used as markers for early disease judgment, for example, the increase in the abundance of *Enterobacter*, *Enterococcus*, *Verrucomicrobium*, *Streptococcus* and *Ruminococcus* and the decrease in the abundance of *Prevotella*, *Blautia*, *Faecalibacterium*, *Roseburia* and *Lachnospira* [205,206,207]. Another study reported an increased abundance of *Verrucomicrobiaceae* and *Firmicutes* and a decreased abundance of *Prevotellaceae* and *Erysipelotrichaceae* in PD patients [208]. The imbalance of these PD-related gut microbiota promoted the progression of PD by increasing intestinal permeability, aggravating neuroinflammation, increasing oxidative stress and reducing neurotransmitter production [209]. Castelli et al. verified that oral compound preparation of *Bifidobacterium* and *Lactobacillus* could effectively protect dopaminergic neurons in PD mouse model induced by 6-hydroxydopamine as well as improve movement disorders [210]. In contrast, the imbalance of gut microbiota may also lead to the impairment of SCFAs production, lipid metabolism, immune regulatory function and intestinal permeability, thus leading to PD [211]. *G. lucidum* extract can regulate mitochondrial function, autophagy and apoptosis by activating AMPK/mTOR and PINK1/Parkin signaling pathways, thus improving the pathological status of PD [212]. *Morchella esculenta* polysaccharides possessed inhibitory activities against acetylcholinesterase and butanoyl cholinesterase, which may be useful for the treatment of PD [213]. However, no studies have evaluated the use of mushroom polysaccharides through regulating gut microbiota for the treatment of PD.

Age-related changes in the gut microbiota can cause brain aging and age-related neurodegenerative diseases. Aging is one of the key factors of a weakened immune system, so maintaining a healthy gut microbiota is crucial to anti-aging. *Lentinan* ameliorated the increase of *Bacteroidetes* and the decrease of *Lactobacillus* and alkali-producing bacteria in the intestinal tract of elderly mice, and partially reversed the composition of age-induced intestinal microbiota by increasing the cytokine levels in the peripheral blood to restore hypoimmunity induced by age [37]. The relative abundance of *Lactobacillus* and *Bifidobacterium* decreased with age [214]. The ratio of *Firmicutes* to *Bacteroides* decreased in older adults in Ireland and France, but this phenomenon was not observed in Italy [215]. *Akkermansia* was more abundant in the gut microbiota of the elderly, suggesting that *Akkermansia* may promote human health and longevity [216].

Excessive fatigue increases the accumulation of harmful metabolites and is a critical health problem [217]. The regulatory effects of *Tuber indicum* on fatigued mice were accompanied by improved intestinal integrity and increased SCFAs. It could decrease the ratio of *Firmicutes* to *Proteobacteria* and the ratio of *Firmicutes* to *Bacteroidetes* in the fatigue group, and elevate the relative abundance of the *Bacteroidetes* and *Actinobacteria* [218].

## 5. Conclusions and Future Perspectives

Polysaccharides are an important active ingredient of mushrooms, which are not absorbed by the gastrointestinal tract and can only be fermented by gut microbiota in the large intestine. Mushroom polysaccharides can also specifically change the composition and abundance of gut microbiota and maintain intestinal microecological balance. It is well known that numerous biological activities of polysaccharides are affected by their complex structure. Therefore, the authors investigated the relationship between the structure of mushroom polysaccharides and its corresponding regulation function of gut microbiota. It was concluded that mushroom polysaccharides containing glucose, galactose, mannose and fucose, as well as (1→3) and (1→6) linkages exerted better regulatory effects of gut microbiota. *Firmicutes*, *Lactobacillus* and *Bacteroides* were the main regulated gut microbiota. However, the exact structure or monosaccharide composition that stimulates specific gut bacteria has not yet been determined. The complex structure and the wide range sources of polysaccharides, and the great differences in the regulation of gut microbiota are the main factors hindering further research in this area. Therefore, it is necessary to strengthen the research on the regulation effect of mushroom polysaccharides specifically targeting gut microbiota. 

In addition, the interactions between mushroom polysaccharides and gut microbiota and their effects on human health were reviewed. It was found that mushroom polysaccharides can promote human health by regulating gut microbiota, promoting the production of SCFAs, improving intestinal mucosal barrier, regulating lipid metabolism and activating specific signaling pathways. Although numerous animal studies have revealed the mechanism of mushroom polysaccharides on disease through the regulation of gut microbiota, further validation and exploration of its potential mechanisms are needed through metabolomics and metagenomics methods and higher-quality clinical trials. Notably, the role of microbial metabolites produced by mushroom polysaccharides in promoting host health is also a major concern to explore, so as to fully understand the multiple benefits of mushroom polysaccharides on human health.

## Figures and Tables

**Table 1:** Effects of the source and structural features of mushroom polysaccharide on the regulation of gut microbiota.

Source	Fractions and Structural Features	Gut Microbiota Modulation	Ref.
*G. frondosa*	GFP: 1.82 × 10^4^ Da; Man/Rha/GlcA/GalA/Glc/Gal/Fuc=25.49/5.18/9.49/7.30/27.59/15.02/9.92	*Alistipes*↑ *Streptococcus*, *Enterococcus*, *Staphylococcus* and *Aerococcus*↓	[20]
*Inonotus obliquus*	IOP: 3.25 × 10^4^ Da; Man/Rha/Glc/Gal/Xyl/Ara=9.80/13.60/29.10/20.50/21.60/5.40	*Bacteroidetes*, *Prevotella* and *Lactobacillus*↑ *Alistipes*, *Incertae_Sedis*, *Helicobacter*, *Parabacteroides* and *Rikenella*↓	[25]
*L. edodes*	L2: 26 kDa; Glc/Gal/Ara=87.50/9.60/2.80	*Proteobacteria*, *Bacteroides acidifaciens*, *Alistipes* and *Helicobacter suncus*↑ *Bacilli*, *Betaproteobacteria*, *Firmicutes/Bacteroidetes*, *Lactobacillaceae* and *Alcaligenaceae*↓	[27,36,37]
*Ramaria flava*	DRFP: 1.02 × 10^5^ Da; Glc/Gal/Man/Fuc/Xyl/Rha/Ara/GlcA=40.61/26.97/17.72/7.78/6.31/0.11/0.06/0.44	*Lactobacillus rhamnosus*↑	[38]
*H. erinaceus*	HEP-30: 8.23 × 10^5^ Da; Fuc/Man/Glc/Gal=0.30/1.30/9.80/0.30; HEP-50: 1.67 × 10^4^ Da; Fuc/Man/Glc/Gal=1.70/0.50/10.60/10.40; HEP-70: 4.77 × 10^3^ Da; Fuc/Man/Glc/Gal=1.20/1.30/23.70/0.30	*Bifidobacterium*, *Faecalibacterium*, *Blautia*, *Butyricicoccus* and *Lactobacillus*↑ *Escherichia-Shigella*, *Klebsiella* and *Enterobacter*↓	[39]
*Helvella leucopus*	p-HLP: 3.91 × 10^9^ Da; Man/Glc/Rha/Gal=43.68/38.16/9.34/4.35	*Verrumicrobiota*, *Lactobacillus* and *Proteobacteria*↑ *Lachnospiraceae genera*, *Lachonospiraceae_NK4A136_group* and *Lachnospiraceae_unclassified*↓	[40]
*L. edodes*	(1, 3)/(1, 6)-β-glucan	*Clostridiales*, class *Clostridia*, family *Lachnospiraceae* and family *Ruminococcaceae*↑	[41]
*Morchella esculenta*	MEP: Man/Glc/Gal/Ara=1.00/14.10/0.61/1.56	*Lactobacillus* and *Firmicutes*↑ *Actinobacteria*, *Corynebacterium*, *Bacteroides* and *Facklamia*↓	[42]
*P. eryngii*	PEP: Man/Rib/Glc/Gal/Ara/Fuc=9.43/0.43/78.32/8.47/3.05/0.30	*Firmicutes*↑ *Proteobacteria* and *Bacteroidetes*↓	[43]
*Sparassis crispa*	SCP-1: 1.36 × 10^4^ Da; Glu/Gal/Fuc/Man=52.10/31.10/15.04/1.76; MC: (1→6)-α-D-Galp, (1→6)-β-D-Glcp, (1→3)-β-D-Glcp, (1→2,6)-α-D-Galp and (1→3,6)-β-D-Glcp; SC: (1→6)-2-OMe-α-D-Galp, (1→4)-β-D-Glcp, (1→3)-β-D-Glcp	*Prevotella 9*, *Dialister*, *Megamonas* and *Megasphaera* ↑ *Escherichia/Shigella*↓	[44,45]
*Tremella fuciformis*	TPs: 2.89 × 10^5^ Da; Man/Rib/Rha/GlcA/Glc/Gal/Xyl/Ara/Fuc=43.68/0.19/0.21/12.93/1.00/0.79/14.40/0.89/11.25; MC: (1→3)-α-D-Manp; SC: D-Xyl, D-Man, L-Fuc, D-GlcA	*Firmicutes/Bacteroidetes*, *Lactobacillaceae* and *Lactobacillus*↑ *Ruminococcaceae* and *Helicobacter*↓	[46]
*Lyophyllum decastes*	LDP1-1: 5.02 × 10^5^ Da; Man/Glc/Gal/Fuc=1.00/2.38/2.58/0.73; DB: 45.90%; 1,3-Fucp, T-Galp, 1,4-Glup, 1,6-Glup, 1,6-Galp, 1,2,6-Manp LDP1-2: 1.13 × 10^6^ Da; Man/Glc/Gal/Fuc=1.00/2.33/2.51/0.78; DB: 43.51%; 1,3-Fucp, T-Galp, 1,4-Glup, 1,6-Glup, 1,6-Galp, 1,2,6-Manp	*Bacteroides intestinalis* and *Lactobacillus johnsonii*↑	[47]
*G. lucidum*	GLP: 1.33 × 10^5^ Da; Glc/Gal/Man/Fuc/Xyl/GlcA=58.97/17.54/8.63/2.79/2.02/6.77	*Bacteroidetes/Firmicutes*, *Bacteroides ovatus* and *Bacteroides uniformis*↑	[48]
*Pleurotus abieticola*	PAPS1: 1.72 × 10^4^ Da; Fuc/Gal/Glc/Man=1.73/49.66/12.00/36.60; MC: →2,6)-α-D-Galp-(1→, →6)-α-D-Galp-(1→ and →3)-β-D-Glcp-(1→; SC: β-D-Manp-(1→ and β-D-Manp-(1→6)-α-D-Galp-(1→	*Prevotella*, *Alistipes*, *Coprococcus* and *Oscillospira*↑	[49]
Wild morels	MP: 3.97 × 10^6^ Da; Man/Glc/Gal/Rha=43.15/19.56/ 20.25 /1.00	*Lachnospiraceae*, *Ruminococcaceae* and *Erysipelotrichaceae*↑ *Lactobacillus*↓	[50]
*G. lucidum*	GLP: 1.37 × 10^4^ Da; Man/Glc/Gal/Rha/Ara=3.16/16.17/3.74/1.65/1.00	*Parabacteroides*, *Adlecreuzia*, *Rothia* and *Bacteroides*↑ *Proteus*, *Corynebacterium*, *Proteus*, *Ruminococcus* and *Coprococcus*↓	[51]
*Auricularia auricular-* *judae* (*Bull.*)	AAP: Rha/Man/Glc=1.46/2.34/0.63	Ruminococcus↓	[52]
*Dictyophora indusiata*	DIP: Glc/Man/Gal=59.84/23.55/12.95	*Firmicutes*, *Clostridia* and *Bacilli*↓	[53]
*Flammuliana velutipes*	FVP1: 5.48 × 10^4^ Da; Glc/Man/Gal=56.20/29.70/14.10	*Lachnospiraceae*, *Bacteroidales family S24-7* and *Firmicutes/Bacteroidetes*↑	[54]
*L. edodes*	LESDF-3: β-D-Arap-(1→, →3)-α-D-Galp-(1→, →3,6)-α-D-Manp-(1→, →4)-β-D-Xylp-(1→, and →2,4)-α-D-Glcp-(1→	*Parasutterella*, *Bacteroides*, *Parabacteroides* and *Lachnospira*↑	[55]
*F. velutipes*	FVP: 7.47 × 10^6^ Da (48.09%) and 1.51 × 10^4^ Da (51.91%); Man/Glc/Xyl/Ara/Fuc	*Allobaculum*, *Lactobacillus*, *Alloprevotella*, *Akkermansia* and *Bifidobacterium*↑	[56]
*Dictyophora indusiata*	DIP: Glc/Man/Gal=59.84/23.55/12.95	*Lactobacillus*↑ *Gammaproteobacteria*↓	[57]
*Auricularia auricular*	AAP: 3.65 × 10^5^ Da; GlcA/Glc/Xyl/Ara/Fuc=7.60/44.20/7.00/35.80/4.50	*Bacteroidetes* and *Porphyromonadaceae*↑ *Firmicutes*, *Lachnospiraceae*, *Rikenellaceae* and *Ruminococcaceae*↓	[58]
*G. frondosa*	GFP: 1.82 × 10^4^ Da	*Helicobater*, *Intestinimonas*, *Barnesiella*, *Defluviitalea*, *Ruminococcus*, *Flavonifractor* and *Paraprevotella*↑ *Clostridium-XVIII*, *Butyricicoccus* and *Turicibacter*↓	[59]
*G. frondosa*	GFP-N: 1.26 × 10^7^ Da; Ara/Man/Glc=3.79/1.00/49.70; →2,6)-α-D-Manp-(1→4, α-L-Araf-C1→, and →3,6)-β-D-Glcp-(1→	*Akkermansia*, *Lactobacillus* and *Turicibacter*↑	[60]
*Dictyophora indusiata*	DIP: Glc/Man/Gal=59.84/23.55/12.95	*Lactobacillaceae* and *Ruminococaceae*↑ *Enterococcus*, *Bacteroides* and *Proteobacteria*↓	[61]
*H. erinaceus*	HECP: 8.67 × 10^4^ Da; Glc/Gal/Ara/Xyl/Rha/Man=76.71/14.26/4.04/2.57/1.32/1.14	*Verrucomicrobia* and *Actinobacteria*↑ *Bacteroidetes*↓	[62]

Note: ↑, increased; ↓, decreased; MC, main chain; SC: side chain; Ara, arabinose; Fuc, fucose; Gal, galactose; GalA, galactonic acid; Glc, glucose; GlcA, gluconic acid; Man, mannose; Ref: reference; Rib, ribose; Xyl, xylose.

**Table 3:** The modulation of the gut microbiota in immunological diseases by mushroom polysaccharides.

Disease	Mushroom	Model	Gut Microbiota Regulation	Effects on Hosts & Functional Mechanisms	Ref.
Chronic pancreatitis	*Inonotus obliquus*	DDC-induced ICR mice	*Bacteroidetes*↑ *Firmicutes*↓	Increased GSH-PX and TAOC levels, and decreased TNF-α, TGF-β, lipase and trypsin levels; reduced the gut microbiota diversity and richness.	[25]
Colitis	*G. lucidum*	DSS-induced Wistar rats	*Ruminococcus_1*, *Pasteurella*, *Fusicatenibacter*, *Enterorhabdus*, *Marvinbryantia*, *Erysipelatoclostridium* and *Anaerofilum*↑	Regulated 11 genes, including six upregulated (Ccl5, Cd3e, Cd8a, Il21r, Lck, and Trbv) and five downregulated (Ccl3, Gro, Il11, Mhc2, and Ptgs) genes; lowered the DAI.	[29]
Colonic injury	*Helvella leucopus*	DSS-induced C57BL/6 mice	*Verrumicrobiota, Lactobacillus* and *Proteobacteria*↑ *Lachnospiraceae_NK4A136_group* and *Lachnospiraceae_unclassified*↓	Downregulated IL-6, IL-1β, TNF-α and COX-2, iNOS; upregulated IL-10.	[40]
Colitis	*Flammuliana velutipe*	DSS-induced SD rats	*Ruminal butyrivibrios*, *Roseburia* and *Bacteroidales family S24-7*↑	Down-regulated inflammatory signal pathways of TLR4\NF-κB; promoted the SCFAs.	[54]
Colitis	*Dictyophora indusiata*	DSS-induced BALB/c mice	*Lactobacillus*↑ *Gammaproteobacteria*, *Proteobacteria*, *Bacteroides* and *Enterobacteriaceae*↓	Dephosphorylated NF-κB and MAPK; inhibited the level of iNOS, COX-2; reduced the level of TNF-α and IL-6.	[57]
Chronic pancreatitis	*G. lucidum*	DDC-induced ICR mice	*Lactobacillales*, *Lachnospiraceae* and *Roseburia*↑ *Prevotella*, *S24-7*, *Bacteroides* and *Helicobacter*↓	Decreased lipase, AMS, IFN-γ and TNF-α level, increased SOD and TOAC; altered the composition and diversity of intestinal microbiota.	[160]
Colitis	*G. lucidum*	AOM/DSS- induced mice	*Bacteroidetes/Firmicutes*, *Lactobacillus and Bifidobacterium*↑ *Oscillibacter*, *Desulfovibrio*, *Alistipes* and *Lachnoclostridium*↓	Downregulated IL-1β, iNOS, and COX-2 expressions; improved gut barrier function; inhibited TLR4/MyD88/NF-κB signaling.	[161]
Colitis	*Lentinan*	LPS-induced Juvenile taimen	*Firmicutes*, *Cyanobacteria*, *Actinobacteria*, *Lactobacillus*, *Bacteroides* and *Brevinema*↑ *Proteobacteria*, *Myroides*, *Klebsiella*, *Raoultella* and *Fusobacteria*↓	Increased the activities of SOD, GSH-Px and CAT, and inhibited the lipid peroxidation; increased the expression levels of TGF-β, TNF-α, IL1β, IL6 and IL8; modified intestinal microbiota.	[162]
Colitis	*H. erinaceus*	TNBS-induced SD rats	*Bacteroides*, *Bifidobacterium*, *Desulfovibrio* and *Lactobacillus*↑ *Corynebacterium*, *Staphylococcus*, *Ruminococcus* and *Dorea*↓	Improved the levels of IL-2, IL-8, IL-10, TNF-γ, TNF-α, VGEF and M-CSF; improved the expression of NF-κB p65, TNF-α, and IL-10.	[163]
Colitis	*Auricularia polytricha* and *F. velutipes*	DSS-induced ICR mice	*Ruminococcaceae, Lachnospiraceae* and *Prevotellaceae*↑	Improved the unbalanced Th1/Th2 and Th17/Treg ratio; inhibited the NF-κB and MAPK/ERK1/2 signaling pathways and stimulated the Keap1/Nrf2 signaling pathways.	[164]
Antibiotic-associated diarrhea	*Antrodia cinnamomea*	ICR mice	*Lachnospiraceae_NK4A136_group*, *Osllospiraceae* and *Lachnospiraceae*↑ *Enterococcus*↓	Reduced the level of TNF-α and IL-6.	[165]
Colitis	*P. eryngii*	DSS-induced ICR mice	*Adlercreutzia*, *Akkermanisa*, *Lactobacillu*, *Anaerostipes* and *Allobaculum*↑ *Turicibacter*, *Dorea*, *rc4-4*, *Bacteroides* and *Prvotella*↓	Reduced the level of IL-1β and IL-17.	[166]

Note: ↑, increased; ↓, decreased; AMS, antimacrophage serum; AOM, azoxymethane; CAT, catalase; COX-2, cyclooxygenase-2; DAI, DNA-dependent activator of IFN-regulatory factors; DDC, Dideoxycytosine; DSS, dextran sulfate sodium salt; ERK, extracellular regulated protein kinase; GSH-Px, glutathione peroxidase; IL-1β, interleukin-1β; IL-2, interleukin-2; IL-6, interleukin-6; IL-8, interleukin-8; IL-10, interleukin-10; IL-17, interleukin-17; IFN-γ, interferon-γ; iNOS, inducible nitric oxide synthase; Keap 1, kelch-1ike ECH-associated protein l; LPS, lipopolysaccharide; MAPK, mitogen-activated protein kinase; M-CSF, macrophage colony stimulating factor; MyD88, myeloid differentiation factor 88; NF-κB, nuclear factor-κB; Nrf2, nuclear factor erythroid 2-related factor 2; Ref, reference; SCFA, short-chain fatty acid; SOD, superoxide dismutase; TAOC, total antioxidant capacity; TGF-β, transforming growth factor-β; TLR4, toll-like receptor 4; TNF-α, tumor necrosis factor-α; TNF-γ, tumor necrosis factor-γ; VEGF, vascular endothelial growth factor.

## Data Availability

Not applicable.
